# The First Case Report in Italy of Di George Syndrome Detected by Noninvasive Prenatal Testing

**DOI:** 10.1155/2015/813104

**Published:** 2015-08-05

**Authors:** Giuseppina Rapacchia, Cristina Lapucci, Maria Carla Pittalis, Aly Youssef, Antonio Farina

**Affiliations:** ^1^Department of Medicine and Surgery (DIMEC), Division of Obstetrics and Gynecology, University of Bologna, Via Massarenti 13, 40138 Bologna, Italy; ^2^Geneticlab, Via Corte Ferrighi 16, Noventa Vicentina, 36025 Vicenza, Italy

## Abstract

Panorama Plus (Natera), a single-nucleotide polymorphism- (SNP-) based approach that relies on the identification of maternal and fetal allele distributions, allows the detection of common aneuploidies and also incorporates a panel of 5 microdeletions including Di George syndrome. We report here the first case of Di George syndrome detected by NIPT in Italy; blood was drawn at 12 weeks' gestation. The patient had an amniocentesis to confirm the diagnosis by MLPA (multiplex ligation-dependent probe amplification) and an ultrasound aimed to detect the features associated with the syndrome. A right aortic arch and suspect of thymus atrophy were detected, but not other severe malformations typical of the disease. The patient terminated the pregnancy at 17 weeks. NIPT allowed an early screening of Di George syndrome. As the patient was at low risk, it is likely that an ultrasound would have missed the condition.

## 1. Introduction

Numerous studies have validated the accuracy of noninvasive prenatal testing (NIPT) using fetal cell-free DNA (cffDNA) to assess the risk of fetal aneuploidies early in pregnancy [1].

cffDNA is the most effective method of screening for trisomy 21, with DR of more than 99% and FPR of about 0.1%. [2–4]. There are two different approaches in analyzing the cffDNA: quantitative and single-nucleotide polymorphism- (SNP-) based methods. The quantitative approach works better for twin pregnancies and donor egg pregnancies, but the SNP-based methods offer a wider spectrum, including the highly sensitive and specific detection of triploidy, vanishing twins, and, so far, 5 microdeletions.

Microdeletion of chromosome 22q11.2 or 22q11.2 deletion syndrome (22q11.2 DS) occurs in 1 : 2000 live births [5]. Up to 93% of cases occur de novo, whereas the remaining 7% of deletion cases are inherited from a parent [6]. Its phenotypic expression is variable and includes Di George syndrome (OMIM 188400) and velocardiofacial syndrome (OMIM 192430). Patients with 22q11.2 deletion syndrome can suffer from congenital heart disease (CHD), cleft palate, velopharyngeal insufficiency with hypernasal speech, hypocalcemia, dysmorphic facial features, and mental retardation [6]. CHD is found in approximately 75% of patients with 22q11.2 DS and typically constitutes conotruncal malformations such as interrupted aortic arch type B, tetralogy of Fallot (TOF), truncus arteriosus communis, and pulmonary atresia with ventricular septal defect (VSD) [7–9].

Even if the samples size of Di George cases collected so far in the various studies is relatively small, the performance of the SNP method is quite impressive. For example, in a study by Wapner et al. [5] the DR for 22q11.2 deletion was 97.8% (45/46) at a false positive rate of 0.76% (3/397). The positive predictive value (PPV) was estimated to be 5.3% based on the incidence of the disease being about 1 : 2000. It is also worth noting that this value is quite similar to that quoted for the combined test as reported in the SURUSS study (1 : 22 or 4.5%) [10]; therefore the potential impact on a routine screening program is very effective.

## 2. Case Presentation

**Figure 1:**
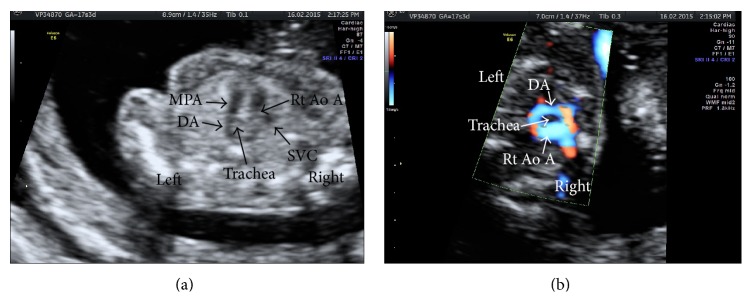
Fetal echocardiography at 17 weeks' gestation. Two-dimensional gray-scale (a) and high definition color Doppler (b) images in three-vessel and trachea view. The right aortic arch (Rt Ao A) and ductus arteriosus (DA), forming classic “U” shape. MPA, main pulmonary artery; SVC, superior vena cava.

A 38-year-old primipara woman was referred for counseling at 15 weeks of gestation because of a high risk finding for Di George syndrome with the estimated risk of 1 : 19 by a SNP-based NIPT performed at 12 weeks' gestation (Panorama Plus, Natera Inc.; San Carlos, California). CffDNA from maternal plasma was amplified at 4,128 SNPs, including 672 SNPs from a 2.91 Mb in the 22q11.2 region, and sequenced. The risk calculation was performed by using the Next-generation Aneuploidy Test Using SNPs (NATUS) algorithm, which involves applying a Bayesian-based maximum likelihood statistical method to the SNP data to determine the number of copies of the 22q11.2 region present in the genome of the fetus, and the likelihood of the determination, expressed as a risk score. Her husband was 45 years old. She and her husband were healthy and nonconsanguineous. There was no family history of congenital malformations. She denied any recent infections or exposure to teratogens during this pregnancy. She was referred to our centre, and amniocentesis was performed for cytogenetic and molecular analysis. DNA extracted from the amniotic fluid was analyzed by MLPA (multiplex ligation-dependent probe amplification) using the SALSA MLPA kit for Di George syndrome, velocardiofacial syndrome, and cat eye syndrome (MRC-Holland, Amsterdam, Netherlands) according to the manufacturer's protocol. The MLPA showed a deletion in the Di George syndrome critical region of chromosome 22 low copy number repeat (LCR). The deletion was determined to have an extension from 2,058 Mb to 3,117 Mb. Ultrasonographic examination was performed at 17 weeks' gestation using a Voluson E8 and RAB 4–8P (4D, 4–8 MHz Convex) probe (GE Healthcare, Milwaukee, WI) which revealed a singleton fetus with right aortic arch (Figure 1). The amount of amniotic fluid and fetal growth were normal. Other internal organs were unremarkable. The fetus did not show any cleft lip or palate. The woman requested a termination of pregnancy (TOP) only after the US scan revealed some of the clinical features of the syndrome.

## 3. Discussion

NIPT for aneuploidy was introduced in Italy a couple of years ago, giving women an alternative to serum screening and ultrasound, with considerably higher sensitivity and specificity to detect trisomies 21, 18, and 13, as well as monosomy X. In 2014, an extended panel for microdeletions NIPT screening was also introduced [5]. Unlike NIPT for aneuploidy, which was an improvement in care, NIPT for microdeletions represents an entirely new offering, as there were previously no methods which could detect microdeletions noninvasively in the first trimester. In fact, at the present, only the invasive search for microdeletions is available and, in Italy, the test is reserved only for those fetuses with an abnormal scan or abnormal nuchal translucency (NT) >3 mm, or in the presence of familiar history, and it is generally performed around 20 weeks' gestation.

This case is the first detection of fetal Di George syndrome in Italy using NIPT in the first trimester. Di George syndrome was detected using a SNP-based NIPT test (Panorama Plus). Panorama Plus uses an algorithm termed NATUS that analyzes up to 28,000 SNPs and determines mutually exclusive probability of a fetal aneuploidy (trisomies 21, 18, and 13 and monosomy X), fetal microdeletion (Di George syndrome, Cri-du-chat syndrome, Prader-Willi syndrome, Angelman syndrome, and 1p36 deletion syndrome) or euploidy. The DR and FPR of aneuploidies [11, 12] and microdeletions [5] have been reported in several studies elsewhere. It is important to note that NIPT is only a screening test and all high-risk results should be confirmed by an invasive, diagnostic testing method.

Multiple options exist for diagnosis of microdeletions from invasive testing samples, such as FISH (fluorescence in situ hybridization), MLPA (multiplex ligation-dependent probe amplification), aCGH (microarray-based comparative genomic hybridization), and next generation sequencing [13, 14]. FISH analyses for microdeletion syndromes have successfully been replaced by MLPA [15–17]. MLPA is a novel, commercially available PCR-based technique analysis. It has several advantages in comparison with FISH analysis: the ability to analyze several loci simultaneously, reliable detection of duplications, and the facility to obtain the result within a similar turnaround time of 1 to 2 days. MLPA is a new technique for multiple quantification of copy numbers at specific target sequences. The ability of Panorama Plus to detect the 22q11.2 microdeletion noninvasively in the first trimester allowed for earlier decision making. In this case, the termination of pregnancy was delayed because the patients chose to pursue additional confirmation by way of an ultrasound scan that revealed a right aortic arch (without an aberrant left subclavian) and a hypoaplasia of the thymus.

## Abbreviations

NIPT: Noninvasive prenatal testing
SNP: Single-nucleotide polymorphism
cffDNA: Cell-free DNA
TOF: Truncus arteriosus communis
VSD: Ventricular septal defect
PPV: Positive predictive value
NATUS: Next-generation Aneuploidy Test Using SNPs
MLPA: Multiplex ligation-dependent probe amplification
TOP: Termination of pregnancy
Rt Ao A: Right aortic arch
DA: Ductus arteriosus
MPA: Main pulmonary artery
SVC: Superior vena cava.
